# Parental involvement in infection prevention and control in low- and middle-income country neonatal units: a scoping review

**DOI:** 10.1186/s13756-025-01643-1

**Published:** 2025-10-14

**Authors:** Lydia Davidson, Chikomborero Kitikiti, Felicity Fitzgerald, Sarah G. Moxon, Alexandra Beedle, Gwendoline Chimhini, Hannah Blencowe, Rudo Chingono

**Affiliations:** 1 London School of Hygiene and Tropical Medicine, MARCH Centre, Keppel Street, London, WC1E 7HT UK; 2 Biomedical Research and Training Institute, The Health Research Institute, Harare, Zimbabwe; 3 Division of Paediatrics, Sally Mugabe Central Hospital, Harare, Zimbabwe; 4 Department of Infectious Diseases, Imperial College London, London, UK; 5 Department of Child Adolescent and Women’s Health, Faculty of Medicine and Health Sciences, University of Zimbabwe, Harare, Zimbabwe; 6 University College London Hosptials NHS Foundation Trust, London, UK

**Keywords:** Family centred care, Infection prevention and control, Neonatal care

## Abstract

**Objective:**

To review the literature on caregiver involvement in infection prevention and control in low- and middle-income country (LMIC) neonatal units (NNUs).

**Introduction:**

There is a high burden and mortality of neonatal infections globally, with most of the burden falling on LMIC. Healthcare-associated infections (HCAIs) are a particular challenge, with neonatal sepsis being one of the most common HCAIs. It is urgent to prevent infections, as both identification and treatment of neonatal sepsis are increasingly difficult in these contexts. Parents are consistently present on NNUs but their involvement in infection prevention and control (IPC) has been underexplored.

**Inclusion criteria:**

Included studies were carried out in LMIC NNUs and reported on caregivers’ involvement in design, implementation or experience of IPC interventions.

**Methods:**

Five databases were searched in four languages and were screened by two authors. Reference searching was carried out of included papers. Data were analysed by each sub-question; caregiver involvement in intervention design (descriptive analysis), caregiver involvement in IPC delivery (quantitative analysis) and caregiver experience of hygiene and care (thematic analysis).

**Results:**

38 studies were included. Caregiver involvement in IPC design was limited, with examples from four papers. 30 papers contained information about caregiver delivery of IPC interventions. Most activities were related to being educated on IPC, carrying out core IPC activities or providing a specific aspect of an intervention (most frequently Kangaroo Mother Care). 10 papers discussed caregiver experience of NNU hygiene including ethnographic accounts from Ghana, Malawi, Mexico, India and Brazil. Across all contexts hierarchical social structures and challenging communication between healthcare professionals and families was a barrier to effective IPC within NNUs. Families showed a good understanding of core IPC practices and an awareness of contextual challenges of IPC.

**Conclusion:**

Caregiver involvement in IPC is limited to date. However, interventions such as Kangaroo Mother Care indicate the benefits that can be achieved. Hierarchical structures and communication challenges between healthcare professionals and families are a barrier to inclusion at present and must be addressed in any designed intervention.

**Supplementary Information:**

The online version contains supplementary material available at 10.1186/s13756-025-01643-1.

## Background

Neonatal infections account for up to 17% of the 2.3 million annual neonatal deaths, with low- and middle-income countries (LMIC) carrying a disproportionate share of the burden [1, 2]. Healthcare-associated infections (HCAI) in neonates are a particular challenge, with those born in LMIC health facilities at 3–20 times higher risk of contracting HCAI compared to those born in high-income country (HIC) settings [3].

One of the most frequent HCAI amongst newborns is neonatal sepsis. Although neonatal sepsis should be treatable, barriers in identifying and managing neonatal sepsis in LMIC frequently result in significant morbidity, mortality and healthcare costs [4]. In resource-limited contexts, the lack of available blood culture for definitive diagnosis and pathogen identification means neonatal sepsis is typically identified and empirical treatment started based on clinical signs [5, 6]. However, this method is often unreliable and with limited information on local antimicrobial resistance (AMR) patterns to guide empiric antibiotic treatment, recommended treatment is often ineffective [7]. AMR is a growing and significant challenge to treating neonatal sepsis. The leading causes of neonatal sepsis in LMICs, Gram-negative bacteria, are commonly resistant to recommended treatment. Evidence of regional variation in AMR further complicates treatment choices [8, 9]. Given these challenges in identifying and treating sepsis, prevention is imperative, particularly in healthcare settings.

However, preventing infections in LMIC neonatal units (NNU) is difficult. High patient to staff ratios place pressure upon staff to prioritise between tasks, with immediately life-saving tasks taking priority [10]. Access to power and water is frequently inconsistent, limiting the ability of staff to carry out essential infection prevention and control (IPC) tasks. Inconsistency in supply can prevent hygiene habits forming, limiting utilisation of resources even when present [11]. In many countries limited investment in IPC at a national level negatively impacts the implementation of recommended practices [12].

Despite the urgency of reducing neonatal infections in healthcare facilities, there are few effective interventions [13]. AMR, the contextual challenges of IPC in the NNU and the limited evidence around effective IPC interventions in these settings increase both the risk of contracting an infection and the associated mortality [3]. It is urgent to identify alternative routes to IPC improvement in LMIC NNUs. One under-explored pathway is collaboration with families. Families are consistently present on NNUs, frequently providing hands-on care for their newborns (e.g. bathing, feeding, changing diapers). Families, however, have limited education on how to provide care within a healthcare setting, where infection risk is higher. Given the absence of formal programming, mothers learn informally from each other [14, 15].

The limited formal involvement of families so far has been shown in a scoping review of IPC bundles of care in LMIC NNUs. This review identified IPC interventions and analysed them by each separate part of an intervention, called an ‘element’. In total only four separate elements that related to parental involvement were found from 295 elements across 44 papers [16]. Within the bundles of care scoping review, the broader involvement of families in IPC in LMIC NNUs was not explored. There is likely to be information on parental involvement outside of bundles of care. This scoping review aims to understand how caregivers have been involved in IPC in LMIC NNUs thus far to inform the development of a collaborative IPC intervention. This review will refer to ‘caregivers’ within the NNU. This acknowledges that caregivers for newborns are predominantly mothers, but can be fathers, other relatives or nominated individuals if the mother is not able to be at the bedside.

The overall aim will be addressed in three sub-questions;

1. How have caregivers been involved in the design of IPC interventions?
2. How have caregivers been involved in the delivery of IPC interventions?
3. What is the caregivers experience of hygiene and care in the NNU?

## Methods

An initial protocol for this study was registered with the Open Science Framework and subsequently developed and published prior to the commencement of the study [17, 18]. The study was carried out in compliance with the Joanna Briggs Institute Manual and reported in accordance with the Preferred Reporting Items for Systematic reviews and Meta-Analyses extension for Scoping Reviews (PRISMA-ScR) Checklist [19, 20].

### Inclusion and exclusion criteria

We included studies carried out in LMIC NNUs that reported on caregivers’ involvement in design, delivery or experience of IPC interventions (see published protocol for details) [17]. Experience was defined as both the directly reported experience by caregivers, but also the perceived experience, as reported by researchers or indicated by surveys where the respondents were not caregivers. All IPC interventions were included, including routine interventions such as hand hygiene.

### Search strategy

Five peer reviewed databases (Medline, CINAHL, Global Health, EMBASE, Web of Science and Global Index Medicus) were searched in four languages (English, French, Spanish and Portuguese) on the 28/02/24. Search terms were developed including MESH terms. Free text and synonyms for the key concepts: ‘*parents*’, ‘*infection prevention and control*’ and ‘*Neonatal Unit*’ and filters were applied for country income level (low- and middle-income) and study type (qualitative and interventional) [17].

### Screening and eligibility

The search results were imported in Endnote 21 and deduplicated. The remaining titles and abstracts were then imported into Rayyan and title and abstract screening was carried out by two independent reviewers (LD and CK). Full texts were reviewed for inclusion by LD and CK, any disagreements were resolved by discussion between LD, CK and GC. Reference searching of included papers was carried out by LD and reviewed by CK.

### Data extraction and analysis

The data were extracted by LD onto a standard extraction form and reviewed by CK. Extracted data included; author, year of publication, country, study type, study aim, stage(s) of research were involving parents, involvement type (e.g. survey, focus group discussion, in-depth interview), parental role in delivering the intervention (e.g. skin care, environmental cleaning) and quotes relating to parental experience of IPC. The three sub-questions were analysed separately;

1. The way in which caregivers were involved in the design of IPC interventions was analysed descriptively using narrative synthesis.
2. The involvement of parents in the delivery of IPC interventions was analysed quantitatively. IPC interventions were separated into individual components and quantified.
3. Direct quotes from caregivers about their experiences of hygiene within the NNU were analysed thematically [21]. Data on perceived experience of caregivers (researchers commentary, or survey data) were analysed narratively.

### Focus of studies on caregivers in IPC

The search identified a high number of studies that held relevant information but were not primarily aimed at answering the questions within this review. Papers were reviewed for how closely they related to questions on caregiver involvement in IPC. To analyse how many papers directly addressed the aims of this review each paper was categorised into one of three levels of focus below.

1. Articles that directly addressed caregivers’ roles in infection control. Caregivers’ involvement in IPC was a stated aim of the study, included in the title or a key finding.
2. Articles that mentioned caregivers’ involvement in IPC, but they were not in the title, a stated aim of the study or a key finding.
3. Studies that include information relevant to caregiver’s involvement in IPC but do not explicitly mention caregivers. e.g. use of a milk bank or studies reviewing implementation of Kangaroo Mother Care (KMC).

## Results

**Fig. 1:**
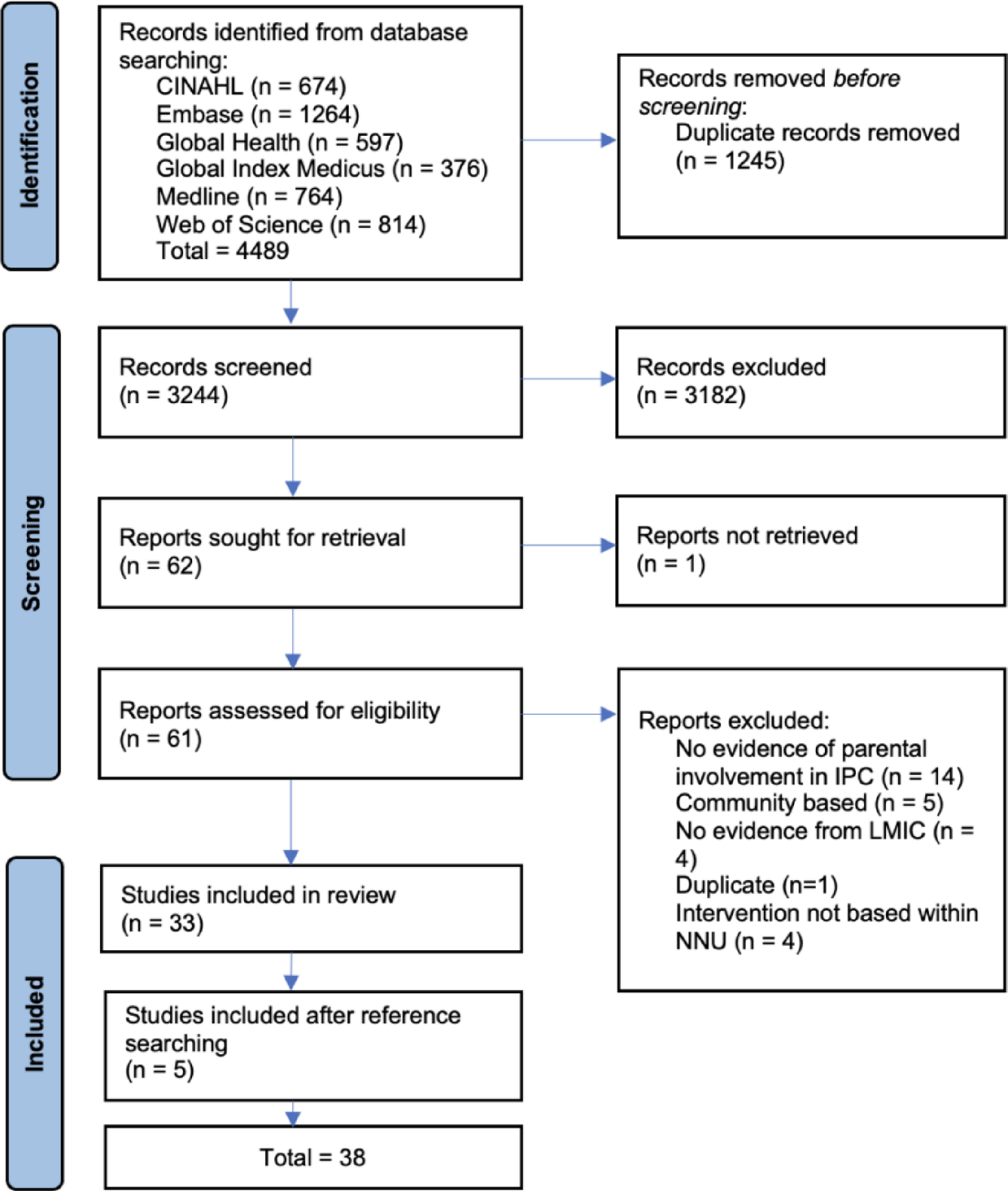
PRISMA flow chart

**Fig. 2:**
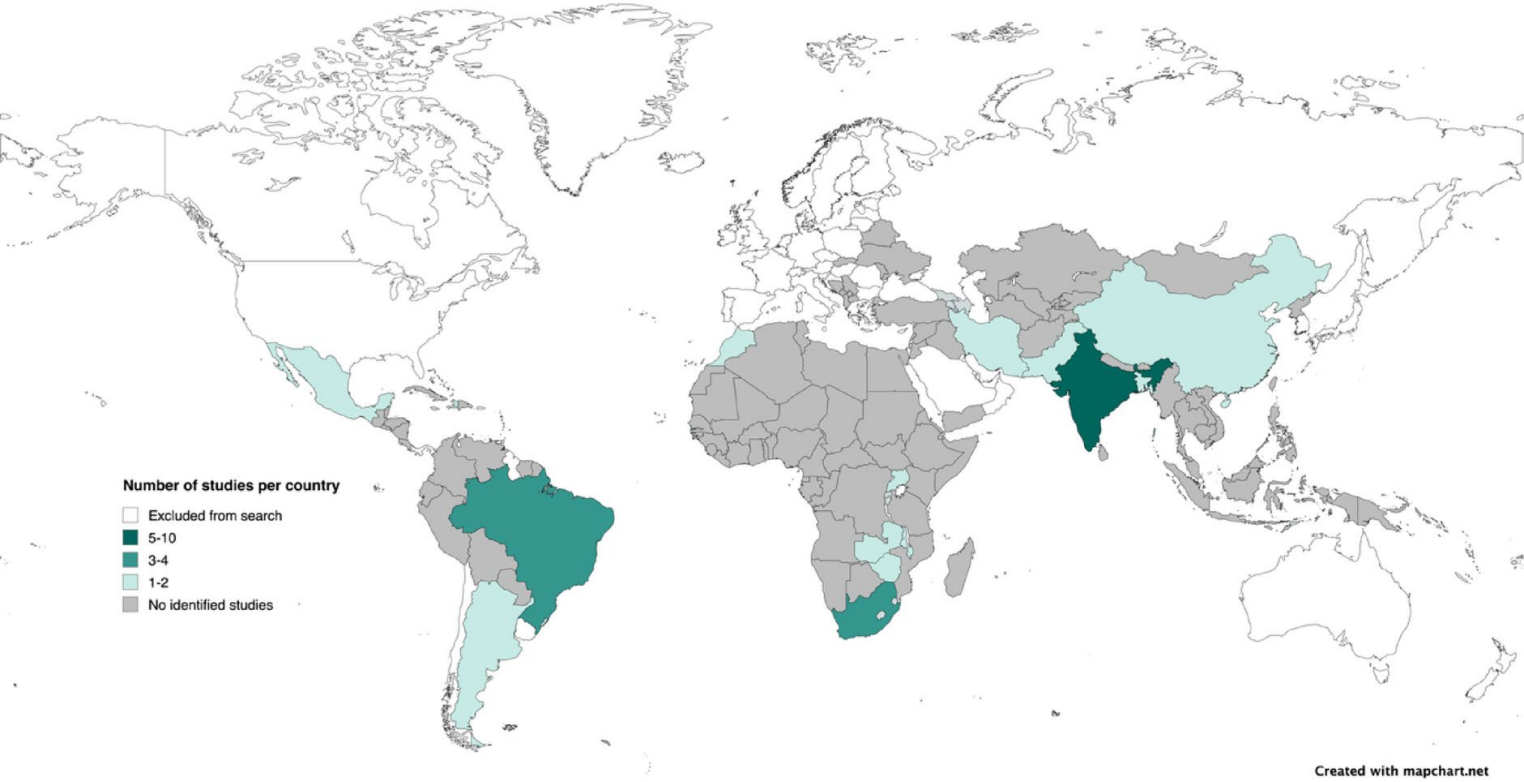
Location of included studies

Thirty-eight studies published between 2005 and 2023 were included (Fig. 1). India (*n* = 10), Brazil (*n* = 4) and South Africa (*n* = 3) contributed the highest number of studies (Fig. 2). Thirteen studies were qualitative and ethnographic, 11 observational (including surveys, cross-sectional, descriptive, chart review), 7 experimental (RCT, quasi experimental), three quality improvement and three cohort studies. 12 out of 38 studies directly addressed caregivers’ roles in infection control with caregiver involvement in IPC as a stated aim of the study, included in the title or was a key finding. 20 of 38 studies discussed caregivers, but were not a stated aim of the study, a key finding or in the title. 6 of 38 studies held relevant information but did not mention parents explicitly. Aims of the included studies were heterogenous including review of specific IPC interventions or key elements of interventions, ethnographic studies reviewing the social context of NNUs, feasibility and acceptability studies and cross-sectional studies.

**Table 1:** Table of included studies by level of focus and sub questions addressed

Study number references (author, year)		Country	Study type	Main aim	Sub-question addressed			Focus
					Q1	Q2	Q3	
1	Agrawaal et al., 2021	India	Quality improvement using PDSA cycles	To improve use of alcoholic hand rub	x	x		2
2	Ahmed et al., 2007	Bangladesh	Prospective survey	To gain insights into the epidemiology, practice, and perceptions regarding oil massage of young Bangladeshi neonates and to assess the acceptability of modified oil-massage practices, including use of skin barrier-enhancing emollients		x		2
3	Karim Sylla, 2021	Morocco	Cross-sectional study	To determine the factors associated to hand hygiene of newborn mothers in the neonatal intensive care unit		x		1
4	Arya et al., 2023	Ghana India Malawi Nigeria Tanzania	Post-hoc analysis of multicentre open-label randomised controlled trial	The objective of this study was post-hoc analysis of the effects of immediate and continuous kangaroo mother care (KMC) on incidence of neonatal sepsis across different weight categories and study sites		x		3
5	Charki et al., 2021	India	Prospective observational cohort study	To identify potential risk factors associated with transmission of neonates born to mothers positive for SARS-CoV-2 at time of delivery and to assess the outcome and thereby elucidate the best infection control clinical practices among mother neonate with COVID-19 infection		x	x	1
6	Chellani et al., 2022	India	Review	This article describes setting up of maternal-neonatal care unit and its experiences including opportunities and challenges and suggests the way forward		x		2
7	Cho et al., 2022	The Gambia	Qualitative study	To understand the perceptions of neonatal healthcare workers toward KMC in unstable neonates < 2 kg in a resource limited, high mortality West African hospital setting		x		3
8	da Silveira & da Silva Dittz, 2021	Brazil	Qualitative – in-depth interviews	To understand the repercussions of social isolation on the daily lives of mothers of babies admitted to the neonatal intensive care unit during the COVID-19 pandemic			x	1
9	Damanabad et al., 2020	Iran	Randomised controlled trial	The aim of this study was to compare the effects of face to face education and video based education on hand hygiene knowledge and performance among mothers in neonatal intensive care unit		x		1
10	Darmstadt et al., 2005	Bangladesh	Randomised controlled trial	To reduce infections in preterm infants less than 33 weeks gestational age through topical therapy with skin-barrier-enhancing emollients		x		2
11	de Almeida Castanho Rozolen et al., 2005	Brazil	Prospective Cohort study	The objective of this study was to verify if the human milk collected at home by breast expression and delivered to the neonatal unit is appropriate for consumption without pasteurization by sick newborns during their hospital stay		x		2
12	De Moura et al., 2020	Brazil	Qualitative – in-depth interviews	To understand the parents’ experience as a strategy for assessing the quality of nursing care in the NNU			x	2
13	Deshommes et al., 2020	Haiti	Quality Improvement	To determine baseline compliance and to improve hand hygiene rates in the St. Damien Hospital neonatal intensive care unit		x		2
14	Dramowski et al., 2017	South Africa	Descriptive outbreak review	This article describes the Tygerberg Children’s Hospital experience with the detection, investigation, and control of outbreaks in the NNU since 2008		x		2
15	Dramowski et al., 2021	South Africa	Propsective, quasi-experimental study	Assessed the impact of a multimodal intervention on the adequacy of surface and equipment cleaning in a South African neonatal ward		x	x	2
16	Geffner et al., 2021	Argentina	Cross-sectional survey	Analyze available resources, guidelines in use, and preparedness to care for newborn infants at maternity centers in Argentina during the COVID-19 pandemic			x	3
17	Herbec et al., 2020	Zimbabwe	Ethnographic study - in-depth interview and participant observations	To explore barriers/facilitators to IPC in a neonatal unit in Harare, Zimbabwe			x	2
18	Karimi et al., 2011	Iran	Descriptive cross-sectional study	To determine the infection level and its relevant organisms as well as to specify drug allergy of the expressed milks from the mothers with their infant admitted to the neonatal intensive care unit		x		2
19	Klingenberg et al., 2021	Multi-country	Cross-sectional survey	To evaluate COVID-19 pandemic preparedness, available resources, and guidelines for neonatal care delivery among neonatal health care providers in low- and middle-income countries (LMICs) across all continents			x	2
20	Magowan et al., 2020	Uganda	Qualitative - in-depth interviews and focus group discussions	To determine the barriers and facilitators to the acceptability of donated human milk for vulnerable infants in this low-income setting from the perspective of caregivers (mothers, fathers and grandparents) as well as healthcare workers		x	x	2
21	Mangochi et al., 2022	Malawi	Ethnographic study - in-depth interview and participant observations	To develop interventions to reduce the transmission of drug-resistant infections based on evidence generated from the study	x	x	x	1
22	Maria et al., 2021	India	Prospective Cohort study	to examine the feasibility and acceptability of the FCC model in a neonatal intensive care unit in India		x		1
23	Mendizabal-Espinsoa & Warren, 2019	Mexico	Ethnographic study - in-depth interview and participant observations	To explore barriers in the inclusion of parents to care for their babies in the neonatal unit		X	x	2
24	Mondkar et al., 2018	India	Qualitative - in-depth interviews and focus group discussions	to understand the perceptions and acceptability of donor human milk and human milk bank among service providers, individual mothers availing services and influencers (fathers and grandmothers) in two health facilities in Mumbai		x		2
25	Murthy et al., 2020	India	Qualitative – in-depth interviews	To explore stressors and support system for families with a neonate admitted with a systemic infection			x	1
26	Olivier et al., 2018	South Africa	Point prevalence survey	To document HCAI rates, antimicrobial use for HCAI, infection prevention staffing, hand hygiene (HH) provisions and HH compliance rates in neonatal and paediatric wards in two district and two regional hospitals in the Western Cape Province, SA		x		3
27	Park et al., 2019	Zambia	Cross-sectional survey	To determine the hygiene practice of mothers of neonatal intensive care unit patients at a tertiary referral centre in Lusaka, Zambia and the factors associated with good hygiene practice		x		1
28	Rodrigues, 2018	Brazil	Qualitative, exploratory case study	To analyze how parents identify patient safety in a neonatal unit.			X	2
29	Salam et al., 2015	Pakistan	Randomised controlled trial	to evaluate the effectiveness of topical applications of coconut oil in reducing the incidence of hospital-acquired bloodstream infection among preterm infants		x		3
30	Sasidharan et al., 2005	India	Quality improvement	To evaluate the outcome of active involvement of mothers/mother substitutes in day-to-day care of high-risk neonates admitted in a level II newborn care unit		x		2
31	Shah et al., 2014	India	Prospective observational study	To evaluate handwashing practices		x		3
32	Suman RP et al., 2008	India	Randomised controlled trial	To compare the effect of KMC and conventional methods of care (CMC) on growth in LBW babies		x		2
33	Sunkwa-Mills et al., 2020	Ghana	Ethnographic study - in-depth interview, focus group discussions and participant observations	To provide data to guide local policy on IPC practices, this hospital ethnographic study examined the factors that influence caregiving in the neonatal intensive care unit, how lay mothers negotiate their roles with health professionals within the hospital context and how these interactions influence the practice of IPC and the reduction of HCAIs	x	x	x	1
34	Sunkwa-Mills et al., 2023	Ghana	Ethnographic study - in-depth interview, focus group discussions and participant observations	To explore the potential to attend to and strengthen care practices in hospital wards	x	x	x	1
35	Verma et al., 2017	India	Randomised controlled trial	To document the impact of structured parental participation of parent attendants in delivery of care to their sick neonates on culture-positive nosocomial infection rate		x		1
36	Vianna, Mayara Sousa, et al. 2012	Brazil	Prospective survey	This study aimed at evaluating the mothers and their companions assimilation of information conveyed during health education activities on the prevention of hospital-acquired infection		x		1
37	Wenani et al., 2023	Uganda	Qualitative – in-depth interviews and focus group discussions	This study aimed to explore the current practices, beliefs and perceptions towards neonatal skin care and to assess acceptability of emollient use with or without massage in eastern Uganda		x		2
38	Yan-Zhi Yi et al., 2021	China	Retrospective chart review	To evaluate the effects of new family centred care strategies and aimed to share our results and experience with other neonatal intensive care units during the COVID-19 pandemic			x	2

Sub-question 1: What is the caregivers’ involvement in IPC intervention design? Sub-question 2: What is the caregivers’ involvement in the delivery of IPC interventions? Sub-questions 3: What is caregivers experience of hygiene and care?

Focus 1 = parents are part of the stated aim of the paper, 2 = parents and IPC are a key finding but not the main aim of the paper, 3 = caregivers are present, but not stated aim or key finding and not explicitly mentioned.

### Q1. Caregiver involvement in IPC design

Four papers contained information relevant to caregiver involvement in IPC design. Three were ethnographic studies that sought to understand the parental experience of hygiene within the context of the NNU as a foundation for later intervention design (Table 1 -*21,31,34*). Two were carried out in Ghana within one NNU and one in Malawi. Caregivers’ experiences of IPC were understood through in-depth interviews, focus group discussions and participant observations. These papers aimed to understand experience as the foundation of later interventions, but the final intervention had not yet been designed. One study included parents as members of the quality improvement team aiming to increase the use of alcoholic hand rub within a NNU (*1*). In this study parents’ insights were key to understanding caregivers’ views of hand rub and increasing use.

### Q2. Caregiver involvement in IPC delivery

30 papers were included that contained information about how parents are involved in the delivery of IPC interventions. Most examples related to caregivers carrying out routine IPC activities (e.g. hand hygiene), being educated on IPC or carrying out care activities for their newborns (Table 2). However, a small number of studies also identified that caregivers were reviewing acceptability of IPC interventions, carrying out laboratory related activities or were involved in outbreak screening as participants. Most studies identified a combination of these different activities.

**Table 2:** Identified components of IPC delivery and in which papers they are identified

Delivery of care	Study number^a^	Number of studies
**Carrying out core IPC activities**	**1**, **3**, **6**, **8**, **13**, **21**, **26**, **31**, **33**, **34**	**10**
Hand hygiene (action rather than being taught)	3, 1, 8, 13, 26, 31, 33, 34	8
PPE – wearing masks	6, 8	2
Provision of bed linen	21	1
**Being educated on IPC**	**5**, **9**, **10**, **13**, **14**, **15**, **22**, **35**, **36**, **30**	**10**
Personal hygiene (handwashing, nail care, bathing)	6, 9, 10, 14, 15, 22, 35, 30	8
Minimum handling of v preterm infants	10	1
Newborn bathing	10	1
Changing diapers	14, 22, 35	3
IPC principles	22	1
Use of surgical masks	5	1
Gowning	10, 35	2
**Care activities**	**4**, **7**, **11**, **18**, **21**, **30**, **32**, **33**	**8**
Feeding	21, 33	2
Changing	21, 33	2
Bathing	21, 33	2
Expressing breastmilk	11, 18, 21, 33, 30	5
Skin-to-skin/Kangaroo Mother Care	4, 7, 32, 33	4
**Provision of part of specific IPC intervention**	**10**, **13**, **15**, **26**, **29**, **31**, **37**	**7**
Provision of emollient	29, 37	2
Hygiene inspections of mothers	10, 13, 15, 26, 31	5
Environmental cleaning	15	1
**Providing review on how acceptable IPC interventions are**	**2**, **20**, **22**, **24**, **37**	**5**
**Laboratory related activities**	**33**	**1**
Convey specimens to laboratory	33	1
Retrieving results	33	1
Purchasing antibiotics	33	1
**As part of outbreak screening**	**14**	**1**

^a^See Table 1 for details of study by study number

### Q3. Caregiver experience of hygiene and care in neonatal units in LMICs

#### Direct reports of experience

10 papers included directly reported experience of parents in relation to hygiene and care. (*8*,*12*,*15*,*20*,*21*,*23*,*25*,*28*,*33*,*34*). Three themes were identified.

1. Emotional impact of hospital IPC measures (8, 23, 25, 28, 33, 34).

Six papers reported on the emotional impact of hospital IPC measures, with fear and anxiety as prominent emotions across contexts.

To take a shower all the time, which I think when I go there, I get scared … I take a shower then I go down, then there are a couple of times that I went there then I go up again to take a shower, because, we don’t know, but every time it passes, you can pass something to us. As she is in the ICU, anything gets infected. So, it is better to go there very clean. (Mother, Brazil). (8)

IPC was shown as a barrier to bonding, with limited parental touch and interactions due to fears of infection transmission and limited visiting times impacting wider family interactions with the newborn.

The most important thing, as the doctor says, is hygiene and when [my son] is very delicate not to touch him, or not to talk to him and try not to make it worse by moving him (Father, Mexico) (23)

Caregivers took on the role of protector of the child, noting situations when hygiene was not carried out as it should be.

I don’t like when the psychologist comes into the unit because she caresses all the babies without washing her hands (Mother, Mexico) (23)

Supportive relationships were also established, with mothers reporting both emotional support and learning between peers within the NNU and gaining further support from both family and staff members.

M: We [mothers] talk. I then understood that many of them have the same problem. In fact, some of the other mothers have more serious problems…Some or the other problem…There are two babies, two months, three months. They have all been admitted. GM: By seeing them, our pain would reduce. All will be talking only about that. All would be having that same pain. It feels like we are all one. (Mother, Grandmother, India) (25)

2. Information imbalance between healthcare workers and families (8, 12, 21, 23, 25, 28, 33, 34).

The reported fear and anxiety was frequently connected to the unknown of the NNU, highlighting an information and power imbalance between staff and families. This was discussed in five papers across three countries (Brazil, Ghana and Malawi).

“What will I ask? I am neither a doctor nor a nurse. I do not understand what is going on” (Mother, Ghana) (34)

“I remember one of the doctors. .he came to tell me the following day. .that my baby was alright. And I thanked him for the information he brought to me, but I still didn’t know where exactly they had sent my baby.” (Mother, Ghana)

“When they come around to do their work, I ask them questions….Some smile and talk to me, others don’t.”(34) (Mother, Ghana)

Hierarchical modes of communication meant that it was difficult to challenge ‘up’ the hierarchy, and ask questions when communication was unclear.

As soon as you wear a patient’s coat, you become a patient. . so sometimes, you wouldn’t want to offend the one taking care of your baby, because you feel that this person is taking care of my baby and what if she leaves my baby? (Mother, Ghana) (33)

Communication was often reported as limited, or sometimes absent.

When entering the NICU there’s a sign that says: Parents have the right to access to information […] but this is only in theory, in practice, that’s not what happens. (Father, Brazil) (28)

Despite this information imbalance, parent frequently displayed knowledge of infection control practice, and how they navigated IPC challenges.

“I just had my own way of extra sanitizing my hands. I pick the chair with my elbow because I don’t want to infect my hands so that I don’t defeat the purpose.” (Mother, Ghana) (34)

3. Competing priorities and practical challenges *(8*,* 21*,* 25*,* 34)*.

Families had competing priorities and additionally showed that they understood the practical challenges within the NNU, and the limitations of the nursing staff practice.

I sometimes feel unprotected, unprotected about thinking about him [son] here at the NICU and also thinking about my loved ones who are at home, who often need to go out to work, that … life still did not stop. (Mother, Brazil) (8)

“Well, judging on the incidents here, when a baby is put on oxygen, and they so happen that the baby has removed the prongs. We call the healthcare workers around, some clean their hands before attending to the baby while others just attend to the babies without doing that because they are in a hurry, I don’t think that’s healthy for the baby but then again, most women really don’t mind as long as their baby has been helped.” (Guardian, Malawi) (21)

Please see Table 3 in the Annexe for a table of themes and supporting quotes.

#### Perceived experience of caregivers

The covid-19 pandemic provided a unique IPC scenario, and many neonatal units altered their visiting patterns. The search returned three studies that surveyed the family involvement in NNUs during the COVID-19 pandemic in Argentina (16), China (38) and 58 LMIC (19). Two were surveys of healthcare professionals and one was a chart review (38). All described increased separation of caregivers and neonates as an infection control measure. Separation of mother and child is widely acknowledged to be a stressful experience for both mother and newborn [22]. These results highlight the increased separation between mother and child, as well as other family members as a response to the covid-19 pandemic and the likely increase in stressful experiences this may have caused.

## Discussion

This review explores the scope of the literature on caregivers’ involvement in the design and delivery of IPC interventions, and their experience of hygiene and care in LMIC NNUs. We found that there is limited literature, with relevant information from 38 papers, but only 12 directly addressing the caregivers’ role. The higher number of papers with a lack of direct focus on caregivers indicates that they are a frequent but underexamined aspect of neonatal care and IPC. Caregivers are rarely involved in the design of IPC interventions, despite being key stakeholders in this context. Caregivers are involved in the delivery of IPC interventions by carrying out core IPC activities such as hand hygiene or use of personal protective equipment (PPE), being educated on IPC or carrying out care activities for their newborns. Their experience of hygiene and care within the NNU is often characterized by fear and anxiety, related to information imbalance between healthcare professionals and caregivers, compounded by poor communication and hierarchy.

Despite the limited literature on caregivers’ roles in IPC, they are constantly present in LMIC NNUs. They are evident as a key part of an intervention (e.g. KMC), evaluating existing interventions (e.g. emollient use), providing care to their infants, carrying out core IPC practices, receiving education, or reporting their experiences of IPC in ethnographic accounts. This complements and adds to initial evidence from a scoping review of IPC intervention bundles in LMIC NNUs, which found three IPC activities that caregivers were involved in; being empowered to carry out routine care, feeding and skin-to-skin care [16]. Despite both reviews providing some evidence on IPC tasks that caregivers carry out in LMIC NNUs, overall evidence remains limited. This is despite it being common practice that caregivers are involved in care for their newborns [15]. Caregivers’ roles within LMIC NNUs may be normalized to the extent that they are rarely directly examined in the context of IPC. However, understanding their experiences and the roles they take on is increasingly understood as key to improving quality of care more broadly in neonatal care [23]. The experiences of caregivers highlighted in this review indicate routes to possible involvement in IPC, balancing between caregiver desire to be involved and not overburdening caregivers with tasks in an already stressful environment.

### Impact of a hierarchical structure on IPC

This review indicates how hierarchical social structures within NNUs negatively impact IPC practices. Studies from Mexico, Malawi and Zimbabwe describe hierarchies with doctors at the top, followed by nurses and caregivers at the bottom [10, 11, 24]. Information asymmetry and power imbalances, particularly affecting caregivers was described in Ghana, India and Brazil [14, 25]. This was shown as an inability to challenge ‘up’ the hierarchy but also in differing expectations of IPC behaviour according to social status. For example, in Mexico parents were expected to be in full personal protective equipment (PPE), but priests were not expected to wash their hands on entrance to the NNU, or between visiting individual patients [24]. In Malawi parents were required to use separate sinks from healthcare workers, and use of hygiene resources by parents was restricted even when adequately supplied [10].

The importance of social structures and behavioural norms in hygiene practices has been acknowledged elsewhere in reference to specific hygiene practices and more broadly to community level water, sanitation and hygiene [26–28]. Understanding the social structure of the NNU is important for effective implementation of IPC interventions. Even when resources are available (e.g. handwashing stations) social norms within the NNU may restrict their use by caregivers, as described in Malawi [10]. Addressing a hierarchical environment aligns with the WHO Multimodal Approach to improving hand hygiene in facilities [29]. This has five separate elements, which in conjunction have been shown to improve hygiene in facilities. The last element is ‘a culture of safety’. Results from this scoping review indicate that addressing power imbalances and hierarchical structures within neonatal units may be a route to building a culture of safety and increasing effective implementation of IPC interventions.

### Perception of caregivers as a risk factor for infection

Caregivers lower position in the NNU hierarchy was compounded by being viewed as a risk factor for infection. This perception was identified both in the types of research that were being carried out, such as surveys identifying risk factors for poor hygiene practices of mothers, as well as how caregivers were discussed within studies. Healthcare workers were concerned about the risk of infection with increased parental presence in the NNU [30, 31]. These concerns were frequently linked to perceptions of caregivers with a lower socioeconomic status or limited education. Specifically, concerns were that caregivers from these backgrounds lacked hygiene literacy and would not be able to understand the technical aspects of IPC due to residing in places without basic sanitation and therefore were at high risk of transmitting infections [14, 24, 32].

In contrast to these perceptions, evidence from ethnographies in this review identified that caregivers frequently had a good understanding of IPC and took efforts to navigate barriers to IPC without formal education or support. This positive indication is also reflected within other interventions, such as immediate Kangaroo Mother Care (iKMC), which has been shown to reduce the risk of mortality due to sepsis by 37% [33]. This reduction is suggested to be directly related to skin-to-skin contact between the mother and child and sharing of the microbiome, confirming active protection against infection by parental contact [34]. One study highlighted the positive impact of including parents in IPC activities and found a reduction in environmental contamination when parents were involved in cleaning their infants cot [35].

While caregivers can contribute to infection transmission, the disproportionate focus may reflect their position within the NNU hierarchy rather than actual risk. The hospital environment is a known reservoir for infection. Pathogens associated with neonatal sepsis have been found throughout the hospital environment, including in hygiene resources such as sinks. One study found healthcare workers hands were more contaminated after handwashing, likely due to sinks being a particular reservoir for infection [36]. Healthcare workers are likely to touch surfaces more frequently than caregivers, and handle multiple patients, therefore also posing a significant risk of infection transmission. Framing caregivers as a separate and significant risk may place emphasis on routes of transmission that are not the highest risk and may miss addressing routes of transmission from healthcare workers. Acknowledging that both parents and caregivers are risks, and taking appropriate measures for both, while addressing hierarchy may lead to more effective implementation of IPC interventions.

## Limitations

There are several limitations to this study. The findings of this study are founded upon published literature. There may be instances where caregivers and clinicians have collaborated more closely on IPC, but this has been part of hospital level quality improvement, rather than research and may not be reflected in the published literature. Additionally, no formal quality appraisal of included studies was carried out. This was not done as the included studies were not only heterogenous in methods but also in aim. Comparing the quality of these studies was unlikely to contribute to the question posed in this review.

## Conclusions

There is limited evidence on caregivers’ roles in IPC in LMIC NNUs to date. However, the range of evidence identified shows their consistent presence and importance. Caregivers are often carrying out IPC-related tasks, such as feeding and nappy changing, but are only occasionally involved in the design or implantation of IPC interventions. The lack of evidence may reflect the normalization of the caregivers’ role within the NNU, rather than a lack of involvement. The caregiver experience in the NNU is dominated by fear and anxiety within a hierarchical system. Addressing this hierarchy and including parents may increase effective implementation of IPC interventions. Additionally, caregivers are often framed as a higher risk of infection in comparison to healthcare workers due to assumptions about their education level and socioeconomic status. However, involving parents has been shown to be beneficial, in environmental cleaning and with interventions such as KMC. This involvement must align with the caregivers’ needs and abilities, as both neonate and caregiver are vulnerable during hospitalisation, and any approach must be sensitive to this.

## Supplementary Information

Below is the link to the electronic supplementary material.

Supplementary Material 1

Supplementary Material 2

Supplementary Material 3

## Data Availability

Publicly available, identified from academic databases.

## Abbreviations

HCAI: Healthcare-associated infection
HIC: High-income country
IKMC: Immediate Kangaroo Mother Care
IPC: Infection prevention and control
KMC: Kangaroo Mother Care
LMIC: Low- and middle-income country
NNU: Neonatal unit
PPE: Personal protective equipment
WHO: World Health Organization
