# Supplementary material for: Parental involvement in infection prevention and control in low- and middle-income country neonatal units: a scoping review
Source: Antimicrob Resist Infect Control. 2025 Oct 14;14:122. doi: 10.1186/s13756-025-01643-1 (PMC12522816; doi:10.1186/s13756-025-01643-1)
Supplement: Supplementary file 1 — Supplementary Material 1 [file 13756_2025_1643_MOESM1_ESM.docx]

*Table 3. Themes and sub-themes of caregivers experience of hygiene and care in LMIC NNUs*

| **Themes** | **Subthemes** | **Illustrative quotes** |
| --- | --- | --- |
| **Emotional impact of hospital IPC measures** *(8, 23, 25, 28, 33, 34)^a^* | Emotional responses | All I feel is tension. I have lost all my happiness. My baby…not sure what may happen. There is panic regarding what may happen to my baby. What is this condition I do not understand…What a horrible mother I must be to have given it to my baby in the womb. No one should go through this. (Mother, India) *(25)*  At one time they say, “[The baby] is getting better, let’s see”. At another time, they say, “There is danger”. Why? What happened suddenly? I am is so frightened. (Mother, India) *(25)*  The doctor or sisters did not inform anything. They just took the baby. How should we understand what is happening inside for so long, and why? I am not telling that I doubt the treatment given or anything. They must be treating properly. But they should tell us something. There is no communication at all. What was wrong with the baby? What are they giving the baby? We just sat there outside waiting anxiously. Some lepsis, what is that? (Father, India) *(25)*  When leaving NICU you are tired and frustrated because you did not finish feeding your baby, and you are wondering if the nurses will continue feeding them for you or not (Mother, Ghana). *(33)*  To take a shower all the time, which I think when I go there, I get scared ... I take a shower then I go down, then there are a couple of times that I went there then I go up again to take a shower, because, we don’t know, but every time it passes, you can pass something to us. As she is in the ICU, anything gets infected. So, it is better to go there very clean. (Mother, Brazil). *(8)*  The word is powerless. Powerless because nothing depends on me, I can't do anything right, I just have to wait, so I feel powerless, that's the right word. (Mother, Brazil). *(8)*  At the hospital, I get more scared because there is a lot of crowding, whether we like it or not, even if it decreases there is, you know. But there is no way. (Mother, Brazil). *(8)* |
|  | IPC as a barrier to bonding | The most important thing, as the doctor says, is hygiene and when [my son] is very delicate not to touch him, or not to talk to him and try not to make it worse by moving him (Father, Mexico) *(23)*  My sister came all the way from Cape Coast (Central region) but she hasn’t seen the baby yet…nobody else has seen the baby, which is worrying (Mother, Ghana). *(33)*  It is difficult because we wanted to have the support of the family, to be able to receive a family member and there is no way because of the virus because I think it is very important for us to have the support of the family, but with this pandemic, it hindered a little. (Mother, Brazil). *(8)*  If I had the father closer, the family closer I think it would be easier for us to go through this difficult time that each one here goes through and is going through. (Mother, Brazil). *(8)*  What has changed is the isolation, like when my boy was born, my mother couldn't come to see him (...) It is the total isolation of the family, it has been a very sad moment for me. (Mother, Brazil). *(8)*  When they brought [me] here, all mothers were with their babies [tears in eyes]. I was the only one staying without my baby here…so painful… [wipes tears] Many mothers without breast milk were keeping their babies [with them]. I had breast milk, but I was unable to keep and feed [my] baby…I felt an uncontrollable rage from within. Many times, I imagined going to the doctor’s room, screaming at [the doctor], secretly taking my baby and running away. [starts crying] (Mother, India) *(25)*  Very difficult, because I went to pick up my daughter after ten days after she was born and even taking her, having contact, I couldn't smell her, hug her as we wanted, kiss her because we have to be alert all the time and protecting them too, who are already in such a delicate situation, within the ICU, it is very complicated. (Mother, Brazil). *(8)*  [...] I don’t keep taking the baby so much, even with all the hygiene care and everything I avoid taking too much. (Mother, Brazil). *(8)* |
|  | Mother as protector | I don’t like when the psychologist comes into the unit because she caresses all the babies without washing her hands (Mother, Mexico) *(23)*  I just had my own way of extra sanitizing my hands. I pick the chair with my elbow because I don’t want to infect my hands so that I don’t defeat the purpose. (Mother, Ghana) *(34)*  While some mothers at the ward have their babies close by, mine is lying so far away. I cannot leave him there and go to sleep, so I sit by his bedside and sleep in the chair if I feel tired. (Mother, Ghana) *(34)*  Some parents come in and out with the gowns, they go into the rooms, they don’t wash their hands. (Mother, Brazil) *(28)* |
|  | Supportive relationships | M: We [mothers] talk. I then understood that many of them have the same problem. In fact, some of the other mothers have more serious problems…Some or the other problem…There are two babies, two months, three months. They have all been admitted. GM: By seeing them, our pain would reduce. All will be talking only about that. All would be having that same pain. It feels like we are all one. (Mother and Grandmother, India) *(25)*  When I would cry, many doctors and sisters have consoled me saying that nothing would happen [to the baby]. I have seen my husband cry when he is alone. I do not want to stress him more by getting emotional in front of him. (Mother, India). *(25)*  For me, I have not started breastfeeding my baby, but sometimes when I see people breastfeeding their babies, I observe (Mother, Ghana) *(34)*  When I came and noticed my baby had been put under the light for phototherapy, I was scared. . . I was confused and was crying until another mother whose baby had previously been put under the light called me, and explained to me that it was going to help my baby. She assured me that my baby was going to be ok (Mother, Ghana) *(34)* |
| **Information imbalance between healthcare workers and families** *(8, 12, 21, 23, 25, 28, 33, 34)* | The unknown of the NNU | What will I ask? I am neither a doctor nor a nurse. I do not understand what is going on (Mother, Ghana) *(34)*  So after the birth of the baby, we will be expecting a healthy baby. We will not be knowing something that is related to all this [infection]…[we] would not be knowing if it would not have happened to us or our parents. We have a belief that [baby care] will happen easily and it will be economical. But when such a thing happens, it becomes a headache, a big problem and heavy burden for people like us, because [we will] not be having any information. (Father, India) *(25)*  We had not heard of anything like this. And never have we seen a small baby having such a condition to be kept in the machine. It was not like this in our times. We have played with many babies and all babies were healthy. We have not seen all of this at all. All our children grew well, we did not have a sight of all this. God should not give anyone this this trouble. (Grandmother, 44 years, neonatal meningitis) *(25)*  GF: Madam, do you know if such babies are admitted here? Does this happen to other babies or is our baby the only one here like this? I want to understand if there are other babies like ours in this hospital…F: Yes, we are very worried, we want to know if this has happened to other babies before. (Father and grandfather, India) *(25)*  Even when I met the doctor the second time, the day before yesterday, I requested, “Sir, will you confirm again that there will be no problem for the baby later? Can you confirm and tell me if there are any other related organs that will be affected afterwards? Please confirm that there will be no problem”. But the doctor told that it cannot be reassured right now and that we may know as the baby keeps growing. But he said that, “95% she is OK, absolutely OK”…But that once concern will persist in the mind that if there is some problem tomorrow…It is very painful. (Father, India) *(25)*  Sometimes they come and tell you that today, your baby will be under the light, but they do not explain why. Is the light supposed to help the baby improve? Is there something wrong? They do not say anything to us. (Mother, Ghana) *(34)*  When I came and noticed my baby had been put under the light for phototherapy, I was scared. . . I was confused and was crying until another mother whose baby had previously been put under the light called me, and explained to me that it was going to help my baby. She assured me that my baby was going to be ok (Mother, Ghana) *(34)* |
|  | Power and information | “I remember one of the doctors. . .he came to tell me the following day. . .that my baby was alright. And I thanked him for the information he brought to me, but I still didn’t know where exactly they had sent my baby.” “When they come around to do their work, I ask them questions.....Some smile and talk to me, others don’t.” (Mother, Ghana) *(34)*  As soon as you wear a patient’s coat, you become a patient. . . so sometimes, you wouldn’t want to o=end the one taking care of your baby, because you feel that this person is taking care of my baby and what if she leaves my baby? (Mother, Ghana) *(33)*  If the one on duty is not doing well with my baby, I will complain; even if the person gets annoyed, I don’t care! (Mother, Ghana) *(33)*  At one time they say, “[The baby] is getting better, let’s see”. At another time, they say, “There is danger”. Why? What happened suddenly? I am is so frightened. (Mother, India) *(25)*  Sometimes they come and tell you that today, your baby will be under the light, but they do not explain why. Is the light supposed to help the baby improve? Is there something wrong? They do not say anything to us. (Mother, Ghana) *(34)*  The doctor or sisters did not inform anything. They just took the baby. How should we understand what is happening inside for so long, and why? I am not telling that I doubt the treatment given or anything. They must be treating properly. But they should tell us something. There is no communication at all. What was wrong with the baby? What are they giving the baby? We just sat there outside waiting anxiously. Some lepsis, what is that? (Father, India) *(25)*  Quote 7: We say ‘Vaidyo Narayano Hari’ (in Sanskrit), meaning ‘Doctor is God’. We have to have faith in them and show our trust. So, we should not question them. Questioning them would be like doubting them, which is incorrect (Father, India) *(25)* |
|  | Difficulties in challenging ‘up’ the hierarchy | As soon as you wear a patient’s coat, you become a patient. . . so sometimes, you wouldn’t want to offend the one taking care of your baby, because you feel that this person is taking care of my baby and what if she leaves my baby? (Mother, Ghana) *(33)*  She (nursing technician) always asked us to wash our hands not to pass on any bacteria. But there were moments when she was on her cell phone and then she got up, didn't wash her hands and touched him (the baby). Then my husband asked: “Don't you have to wash your hands? Don't you have to wear a glove?” - “No, I already did.” But I think it was a slight negligence, but he (the father) got worried. (Mother, Brazil) *(12)*  She had concerns about the safety of the second twin and stated: If the one on duty is not doing well with my baby, I will complain; even if the person gets annoyed, I don’t care! (Mother, Ghana) *(33)*  One outspoken mother mentioned that some of the nurses found her irritating for being rather assertive: I mean once I find out the thing is not properly handled, I am not going to tolerate that, so they felt that I was irritating (Mother, Ghana) *(33)*  I don’t like when the psychologist comes into the unit because she caresses all the babies without washing her hands (Mother, Mexico) *(23)* |
|  | Communication – limited absent or misunderstood | When entering the NICU there’s a sign that says: Parents have the right to access to information [...] but this is only in theory, in practice, that’s not what happens. (Father, Brazil) *(28)*  I take a lot of interest in what is going on. I ask a lot of questions...I get very interactive with the nurses when they are not busy...if they are busy, I don’t stress them... I can imagine their frustrations...I mean if they can’t answer my questions at the time, they would get back to me later. (Mother, Ghana) *(34)*  They [doctors] are saying now that the intestines are not working…and it’s started vomiting now. Something has happened. They are not telling us, something has suddenly happened. (Grandfather, India) *(25)*  I am waiting when I can go back. But I am not getting any information on when my baby will get better. They have been trying for quite a while. They say that there is problem in the…what do you call that? (points to the throat). They don’t seem to know. They did some procedures, they even got a surgeon here to assess. But they are not sure what it is. They are trying different things. I want to be certain about my baby…(Father, India) *(25)*  We need the doctor to come by more every day and talk to us [...] I think they use very technical terms. (Mother, Brazil) *(28)* |
|  | Knowledge of parents | I just had my own way of extra sanitizing my hands. I pick the chair with my elbow because I don’t want to infect my hands so that I don’t defeat the purpose. (Mother, Ghana) *(34)*  I believe that in some cases it’s because we do not have a choice but, you’d see two or more babies sharing a bed. The two babies may have different cases, but since they are being kept in the same place, it is very easy for them to share infections with each other. If there was a way that every baby should be put on their own place, that would prevent them from sharing infections to one another. (Female Guardian, Malawi) *(21)*  She (nursing technician) always asked us to wash our hands not to pass on any bacteria. But there were moments when she was on her cell phone and then she got up, didn't wash her hands and touched him (the baby). Then my husband asked: “Don't you have to wash your hands? Don't you have to wear a glove?” - “No, I already did.” But I think it was a slight negligence, but he (the father) got worried. (Mother, Brazil) *(12)*  Some parents come in and out with the gowns, they go into the rooms, they don’t wash their hands. (Mother, Brazil) *(28)*  So, you have to wash your hand, wear your mask, and avoid hanging around here in the hospital. (Mother, Brazil) *(8)*  Mask all day, wash your hand all the time, every time we go to eat, every time you go to touch the child [...] the utmost care, of course. And you have to change the mask, not staying with the mask for a long time, taking turns. (Mother, Brazil) *(8)*  Something that I would not have ... that I am not used to doing before the pandemic is to wash my hands all the time, using alcohol, a mask ... like this. Even with my clothes, clothes that go on the street, I don't go to the ICU. (Mother, Brazil) *(8)*  Each baby must have their own thermometer, each baby must have their own things. (Mother, Brazil) *(28)*  You need to wash your hands, you must practice hygiene. (Mother, Brazil) *(28)*  If we don’t wash our hands before and after caring for the baby, we can put the baby at risk. (Female Guardian, Malawi) *(21)*  I asked the doctor... but they are sleeping in the same incubator...they touch each other? She said...‘ok yes, ideally, we should have moved one out when we realized the other had an infection’...I was like...let’s do the test on the other one and see if he’s fit. They did the test and he had also picked up the infection. So now they are both on the Vancomycin (Mother, Ghana) *(33)*  I can help my baby to be safe from infection by cleaning properly. (Mother, South Africa) *(15)*  I think they can have another place for us the mothers to breastfeed instead of being in the same room with the incubators and other machines...the place is small, and the mothers are many...we can even spread infection to the babies. (Mother, Ghana) *(34)*  When our baby was on ventilator, and regardless of the critical issues, (doctors) should at least initiate mother’s milk. Mother’s milk, everyone knows, is next to amrut [immortality potion]. It is God’s gift. There is no replacement for mother’s milk, even if you give a NASA scientist-prepared milk. (Father, India) *(25)* |
| **Competing priorities and practical challenges** *(8, 21, 25, 34)* | For parents | I sometimes feel unprotected, unprotected about thinking about him [son] here at the NICU and also thinking about my loved ones who are at home, who often need to go out to work, that ... life still did not stop. (Mother, Brazil) *(8)*  Sometimes I get worried here, as I have other kids out there, I get worried about them too. There it is, divided between them. (Mother, Brazil) *(8)*  First, I'm avoiding going to my house, right, because I have three more children besides the one here, I have three more children, two of whom are still small. (Mother, Brazil) *(8)*  Over here you don’t take something for someone. You can’t take anything for someone, even if it’s a cot sheet...even if you want something, you have to tell the nurse, especially with the cups that we express the milk into. They take the cup themselves, you don’t have to touch it (Mother, Ghana) *(34)*  I could not come here immediately when this happened. I cannot just leave everything and come suddenly. I have to make lot of adjustments at work…I have to work till evening and then I come with home food for my wife. I stay the night and leave in the morning for work. It has been 3 days I have not seen the baby, because visiting hours are closed by the time I can come. Maybe Sunday I can see (Father, India) *(25)* |
|  | Parents awareness of nursing challenges | Well, judging on the incidents here, when a baby is put on oxygen, and they so happen that the baby has removed the prongs. We call the healthcare workers around, some clean their hands before attending to the baby while others just attend to the babies without doing that because they are in a hurry, I don't think that's healthy for the baby but then again, most women really don't mind as long as their baby has been helped. (Female Guardian, Malawi) *(21)*  I believe that in some cases it’s because we do not have a choice but, you’d see two or more babies sharing a bed. The two babies may have different cases, but since they are being kept in the same place, it is very easy for them to share infections with each other. If there was a way that every baby should be put on their own place, that would prevent them from sharing infections to one another. (Female Guardian, Malawi) *(21)*  I take a lot of interest in what is going on. I ask a lot of questions...I get very interactive with the nurses when they are not busy...if they are busy, I don’t stress them... I can imagine their frustrations...I mean if they can’t answer my questions at the time, they would get back to me later. (Mother, Ghana) *(34)*  I find myself being nosy...I would like to know when they are administering the medication...when I come, I want to follow up on it...please, did you give this medication at this time to my baby? There have been more than four occasions when it hasn’t been administered...because we are many, and probably...they forgot...but once you remind them, they do it. (Mother, Ghana) *(34)* |

**^a^** See table 1 for details of study by study number
